# High-resolution 3D whole-heart bright- and black-blood imaging with co-registered T2 mapping at 0.55 T

**DOI:** 10.3389/fcvm.2025.1572318

**Published:** 2025-07-03

**Authors:** Ivan Kokhanovskyi, Carlos Castillo-Passi, Michael G. Crabb, Carl Ganter, Simon J. Littlewood, Karl P. Kunze, Dimitrios C. Karampinos, Marcus R. Makowski, Daniel Rueckert, Claudia Prieto, René M. Botnar

**Affiliations:** ^1^ Institute of Diagnostic and Interventional Radiology, School of Medicine and Health, Technische Universität München, Munich, Germany; ^2^ Institute for Advanced Study, Technische Universität München, Munich, Germany; ^3^ School of Biomedical Engineering and Imaging Science, King’s College London, London, United Kingdom; ^4^ Millenium Institute for Intelligent Healthcare Engineering, Santiago, Chile; ^5^ School of Engineering, Pontificia Universidad Catolica de Chile, Santiago, Chile; ^6^ Institute for Biological and Medical Engineering, Pontificia Universidad Católica de Chile, Santiago, Chile; ^7^ MR Research Collaborations, Siemens Healthcare Limited, Camberley, United Kingdom; ^8^ Chair for AI in Healthcare and Medicine, Technical University of Munich (TUM) and TUM University Hospital, Munich, Germany; ^9^ Department of Computing, Imperial College London, London, United Kingdom

**Keywords:** low-field MRI, 3D whole-heart, simultaneous bright-and black-blood, high-resolution coronary angiography, T2 mapping

## Abstract

**Introduction:**

Conventional CMR exams for assessment of cardiac anatomy and tissue characterization require multiple sequential 2D acquisitions under breath-hold in different orientations, in addition to being limited to 1.5 T and 3 T.

**Methods:**

In this study, we sought to develop a novel 3D motion-compensated free-breathing sequence for comprehensive high-resolution whole-heart assessment of cardiovascular anatomy via simultaneous bright- and black-blood imaging and co-registered $T_{2}$ myocardial tissue quantification in a one-click scan at 0.55 T.

**Results:**

Good agreement with a spin-echo reference sequence was found in the phantom for $T_{2}$ mapping. In-vivo, the proposed research sequence was evaluated in 10 healthy subjects, providing great delineation of cardiac and vascular structures, good visibility of coronary arteries and accurate $T_{2}$ parametric mapping in a clinically feasible time of less than 9 min.

## 1 Introduction

Cardiovascular diseases (CVDs) are the leading cause of mortality, responsible for a third of all deaths globally (1). Efficient screening is a key factor in the early detection of CVDs. Cardiovascular magnetic resonance (CMR) imaging is a well-established non-invasive imaging modality for a comprehensive evaluation of the structure and function of the heart, without the inherent risks associated with the exposure to ionizing radiation. In addition, quantitative myocardial tissue characterization with $T_{1}$ and $T_{2}$ mapping provides valuable information on the presence of myocardial fibrosis, edema, or inflammation, especially in CVDs with diffuse tissue alterations (2, 3). However, CMR examinations remain costly and complex procedures with restricted access for a significant part of the global population, especially in low- and middle-income countries (4).

With the recent introduction of high-end low-field MRI scanners, there is great potential to make CMR more accessible and affordable. Compared to mid- and high-field MRI, the installation and maintenance costs of low-field units are significantly reduced, in addition to lower weight and smaller size of low-field MRI scanners (5, 6). Overall patient comfort is improved due to the wider bore opening and reduced vibration of gradients (7). To address claustrophobia concerns, low-field magnets can be configured in open geometry systems, expanding the ability to integrate stress testing into CMR examinations. The specific absorption rate (SAR) scales with $B_{0}^{2}$, resulting in reduced tissue or device heating in low-field MR scanners (8). Off-resonance and susceptibility artefacts is also reduced, enabling near-device imaging (e.g., pacemakers, sternal wires) and spiral trajectories for efficient diagnostic imaging (9).

Despite the aforementioned advantages, low-field applications still face some limitations, preventing their daily clinical use (10). Selecting the optimal scan parameters requires a trade-off between clinically acceptable scan times, image resolution and quality. Especially for low-field CMR systems with limited gradient performance, new methods for both image acquisition and reconstruction are needed (11). One of the main challenges of low-field MRI is the lower signal-to-noise ratio (SNR), which scales with $B_{0}^{3/2}$, resulting in images with reduced spatial resolution or requiring longer scan time (12). In addition, motion correction algorithms successfully used at higher field strengths may be challenging to adopt at low-field due to the lower SNR of the source images. Favorable changes in relaxation parameters, with a significantly shorter $T_{1}$ and a slightly longer $T_{2}$ (13), at lower field strength partially compensate for SNR loss due to slower signal decay and faster magnetization recovery, which may render parametric mapping more time efficient. Balanced steady-state free precession [bSSFP (14)] sequences offer excellent SNR efficiency and high blood-to-myocardium contrast and can therefore help to compensate for the SNR disadvantage. Moreover, typical bSSFP banding artefacts caused by $B_{0}$ inhomogeneity are less pronounced in low-field scanners, making it a promising choice for anatomical imaging (15).

There is still limited experience with high-resolution anatomical imaging and parametric mapping of the heart at low-field strengths, especially when performed in a single scan. Typical clinical CMR protocols require complex planning by expert personnel, involving several sequential 2D acquisitions in different orientations with multiple breath-holds, leading to long and unpredictable examination times (16). To overcome the above limitations, a combined bright- and black-bood 3D imaging sequence was developed for simultaneous lumen and wall visualization of the great thoracic vessels and cardiac structures (17–19). This was further adapted to enable a combined anatomic imaging and $T_{2}$ tissues quantification at 1.5 T (20). The feasibility of combined bright- and black-blood imaging has been recently demonstrated at low-field (21). In this study, we sought to develop a novel motion-compensated free-breathing CMR research sequence at 0.55 T that allows simultaneous 3D whole-heart assessment of cardiovascular anatomy via bright- and black-blood imaging and $T_{2}$ myocardial tissue quantification in a single one-click scan within a clinically feasible scan time of less than 9 min.

## 2 Materials and methods

### 2.1 Pulse sequence framework

**Figure 1:**
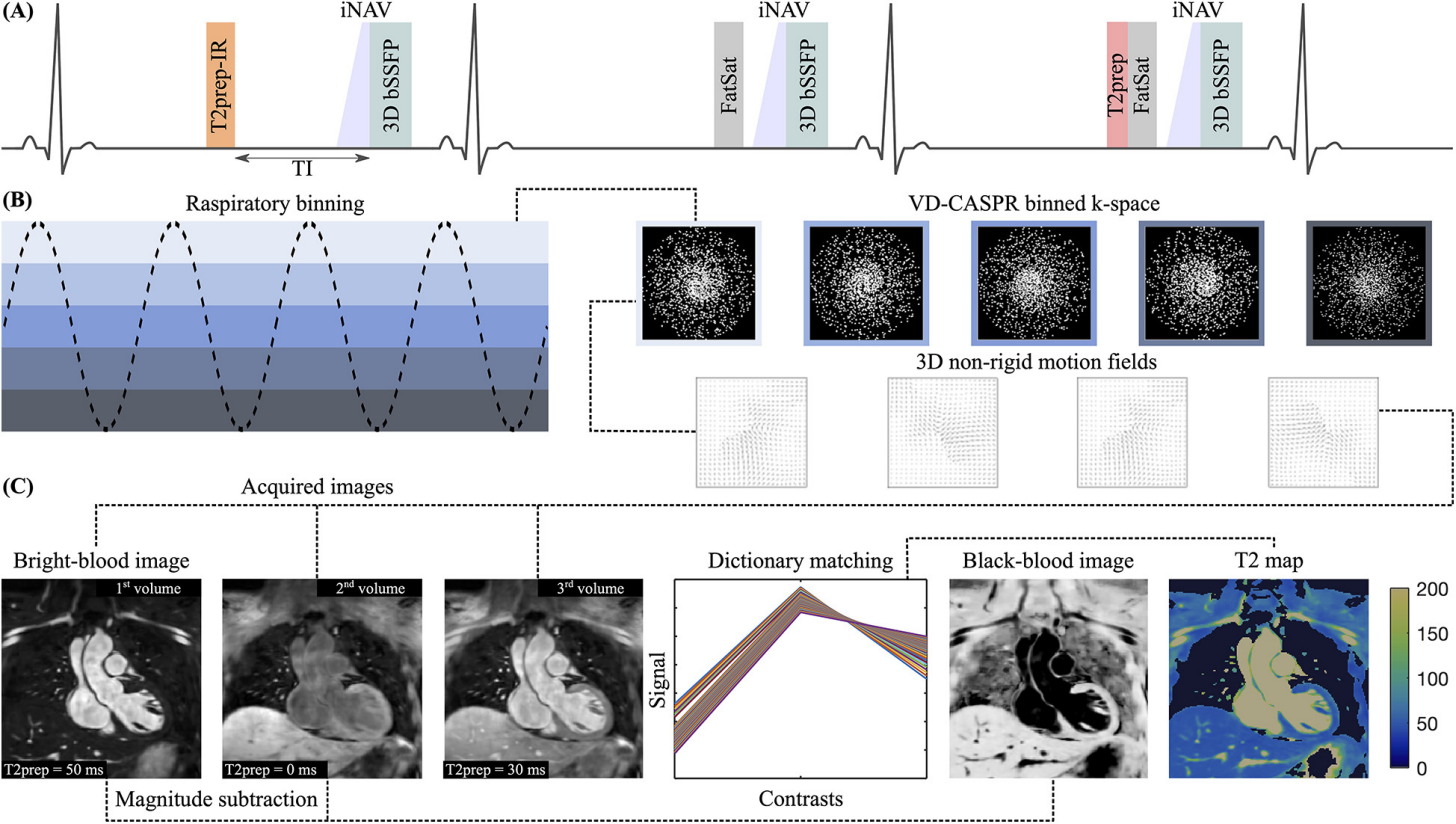
(**A**) Sequence diagram over three interleaved heartbeats (HBs): $T_{2}$prep (50 ms) IR (TI = 90 ms)—none—$T_{2}$prep (30 ms). (**B**) Respiratory binning of data acquired with VD-CASPR to derive 3D non-rigid motion fields. Images are reconstructed in-line using motion-corrected itSENSE followed by off-line HD-PROST for denoising. (**C**) Contrasts are shown for the mid-coronal slice. 3D datasets for each volume can be found in the Supplementary Material. The bright-blood image is acquired in the 1nd HB, while the black-blood image is obtained through direct magnitude subtraction of the 1st from the 2nd volume. $T_{2}$ maps are generated voxel-wise by dictionary matching.

The proposed ECG-triggered research sequence consists of a repeating set of three heartbeats (HBs) arranged in an interleaved fashion, incorporating an adiabatic $T_{2}$ preparation ($T_{2}$prep) module accompanied by an inversion recovery (IR) pulse in the 1st, no preparation in the 2nd and another $T_{2}$prep in the 3rd HB (see Figure 1A). Frequency-selective fat saturation (FatSat) pulses are employed to suppress epicardial fat in the 2nd and 3rd HBs. In the 1st HB, fat suppression is achieved by IR pulse with an appropriate inversion time (TI). 3D bSSFP acquisition is performed in coronal orientation during the mid-diastolic rest period to minimize cardiac motion by adjusting a subject-dependent trigger delay. 2D low-resolution image-based navigators (iNAVs) are acquired prior to each HB by spatially encoding ramp-up pulses to perform beat-to-beat intra-bin translational food-head and left-right motion estimation and correction, as well as respiratory binning of data (see Figure 1B) (22). A variable density 3D Cartesian spiral-like k-space trajectory with a golden angle step (VD-CASPR) is employed to collect a highly undersampled data with 100% respiratory efficiency (23). The 1st volume serves as a bright-blood image, while a black-blood volume is obtained by direct magnitude subtraction of the 1st from the 2nd volume (see Figure 1C). Co-registered $T_{2}$ maps are generated voxel-wise by dictionary matching of all three volumes.

### 2.2 Imaging parameters

$T_{2}$prep modules with hyperbolic-secant adiabatic-refocusing RF pulses of 50 ms/30 ms in duration, Gaussian shaped FatSat pulses with an optimized flip angle of $180^{\circ }$ and 26.6 ms in duration (24), TI=90ms, flip angle (FA) of bSSFP readout = $110^{\circ }$, TR/TE=4.90ms/2.45ms, 30 segments (lines in k-space), acquisition window ≈150ms, 6 ramp-up pulses (also used for low-resolution iNAV spatial encoding), field of view (FOV) = 312 × 312 × 108–120 ${\mathrm{mm}}^{3}$, isotropic spatial resolution = 1.5 mm, VD-CASPR trajectory with an acceleration factor of 4.

### 2.3 Experimental protocol

The proposed framework was evaluated on the standardized cardiac T1MES phantom (25) and 10 healthy subjects (27±3 years, 3 females, heart rate (HR) of 55–78 bpm, body mass index (BMI) of 23–30 $\mathrm{kg}/m^{2}$). All data was acquired on a commercially available 0.55 T MRI scanner (MAGNETOM Free.Max, Siemens Healthineers, Forchheim, Germany) using a 6-channel small flexible coil and 9-channel spine coil, with an external ECG monitoring system (Maglife Serenity, Schiller) connected to the external trigger input. In the phantom experiment, the sequence framework was validated against a 2D IR spin-echo (SE) research sequence with SE times between 6.7 ms and 267 ms. The mean R-wave peak to R-wave peak (RR) interval for each healthy subject was extracted from an axial CINE acquisition acquired at the start of the study to obtain HR=60/RR[s]. Before undergoing the examination, written informed consent was collected from all volunteers.

### 2.4 Image reconstruction & denoising

Images were reconstructed using 3D non-rigid motion-corrected iterative sensitivity encoding [SENSE (26)] performed directly on the scanner in-line, providing magnitude datasets for each of three volumes separately (27). Subsequently, images were denoised using patch-based low-rank regularization [HD-PROST (28)], which groups similar patches in the multi-contrast images. The following parameters were used for HD-PROST: singular value decomposition (SVD) threshold = 0.1, voxel search window 20×20×20, voxel patch-size 6×6×6, number of selected similar patches = 20, and a voxel patch-offset = 5 when searching for similar patches.

### 2.5 Dictionary generation & matching

The numerical simulation of expected signals was implemented in MATLAB (The MathWorks Inc, Natick, MA, USA) using the extended phase graph [EPG (29)] method. To achieve a steady-state magnetization, the simulation involved two sets of dummy sequence repetitions without any signal sampling. The signal evolution from the third repetition was used to generate a unique, HR-specific three-point dictionary. The dictionaries were generated for a fixed $T_{1}=700\;\mathrm{ms}$, corresponding to a typical value of normal myocardium, with various $T_{2}$s in the range [20:2:40,41:1:50,50.5:0.5:60,61:1:70,72:2:90] ms ([lower value: step size: upper value])) to cover relevant values for healthy ($T_{2}=58\;\mathrm{ms}$) and abnormal ($T_{2}=70\;\mathrm{ms}$) myocardium at 0.55 T. A short dictionary for the arterial blood with $T_{1}=1100\;\mathrm{ms}$ and $T_{2}$s in the range [95:5:250] ms was added to better separate a pool of blood from the myocardial tissue. In the phantom experiment, dictionaries with fixed mean $T_{1}$ values were generated for each vial with $T_{2}$s corresponding to myocardium- (A–F) and blood-like (G–I) range (see Table 1). $T_{2}$ parametric values were matched voxel-wise by minimizing the least squares error (LSE) cost function between the normalized measured signal and the dictionary generated entry over three points.

**Table 1:** T1MES phantom containing 9 agarose-based tubes with relevant cardiac $T_{1}$ and $T_{2}$ combinations to mimic myocardium- and blood-like material. The $T_{1}$s were measured with the IR SE and $T_{2}$s with SE multi-echo without IR preparation research 2D sequences at 0.55 T (see Section 2.3) and evaluated in MATLAB.

Mimic material	Phantom vial	Reference T1 [ms]	Reference T2 [ms]
Myocardium	A	424±3	47.7±0.7
	B	543±3	49.3±0.7
	C	300±2	47.4±0.6
	D	994±4	52.9±0.7
	E	1183±8	53.6±0.8
	F	751±4	52.9±0.7
Blood	G	450±4	197±2
	H	1461±14	238±4
	I	265±2	174±2

### 2.6 Data analysis

Anatomical image quality was measured by visibility, which is defined as the normalized signal difference between myocardium and blood signal from the 1st volume for the bright-blood image and from the 2nd–1st subtracted volume for the black-blood image. Different orientations in short-(SAx), vertical (VAx) and horizontal (HAx) long-axis views were obtained in the DICOM viewer Horos (Horos Project, Geneva, Switzerland). Multiplanar reformation and quantitative analysis of individual coronary vessels was performed using Soap-Bubble (30). Image quality was graded using the following scale (31): 0, non-diagnostic; 1, poor (limited coronary vessel visibility or noisy image); 2, average (coronary vessel visible but diagnostic confidence low); 3, good (coronary artery adequately visualised and image of diagnostic quality); and 4, excellent (coronary artery clearly depicted). The scores were assigned by a cardiology clinical research fellow with three years of experience in CMR. For quantitative analysis, vessel sharpness (VS) was calculated as the magnitude of the local change in signal intensity using a first-order derivative (32) (VS of 100% refers to a maximum change in signal intensity at the vessel border, a lower value is consistent with inferior vessel sharpness) over the first 4 cm along the right coronary artery on a bright-blood contrast. To assess the performance of the $T_{2}$ mapping, the correlation plot and Bland-Altman analysis were carried out for axial images of the tubes in the phantom. The bias was adjusted by varying the amount of k-space line segments used for dictionary matching in the range [2:2:12] ([lower value: step size: upper value]) out of 30. To visualize in-vivo data, bull’s-eye plots were generated from 16-segment 3D $T_{2}$ maps according to the American Heart Association model (33) (without apical segment) using the 30 slices acquired along SAx from apex to basal regions (corresponding to a length of 4.5 cm). The mean and standard deviation (SD) of the $T_{2}$ values were calculated for each segment separately. The accuracy of the proposed method was determined using the coefficient of variation (CoV), defined as the SD divided by the mean value. To estimate spatial variability, $T_{2}$ values for the apex, mid-cavity and basal areas were compared across all healthy subjects. The mid-cavity SAx slice was used for visual comparison.

## 3 Results

### 3.1 Sequence design optimization

**Figure 2:**
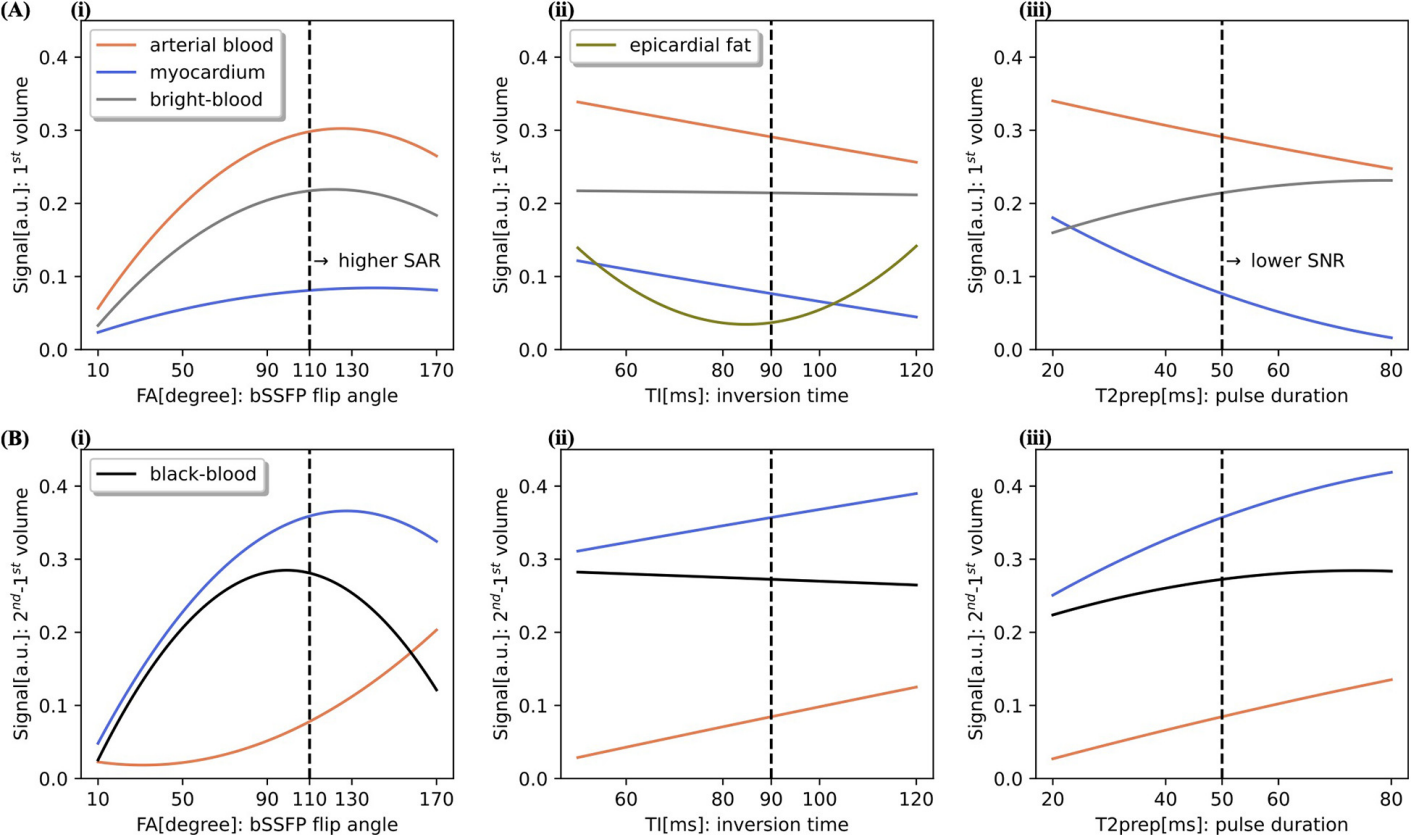
Optimization on sequence parameters using EPG simulations at 0.55 T for a HR of 60 bpm. Simulated signals of the arterial blood ($T_{1}=1100\;\mathrm{ms},T_{2}=260\;\mathrm{ms}$), healthy myocardium ($T_{1}=700\;\mathrm{ms},T_{2}=58\;\mathrm{ms}$) and epicardial fat ($T_{1}=180\;\mathrm{ms},T_{2}=93\;\mathrm{ms}$) are shown for the 1st volume (**A**) and 2nd–1st subtracted volume (**B**). Contrast values for the bright- and black-blood are defined as a difference between the corresponding signals. In each graph one of the following parameters was varied, while the other two were kept constant: imaging flip angle (FA), inversion time (TI) and $T_{2}$prep pulse duration. Signal dependency on (i) FA with fixed inversion TI of 90 ms and $T_{2}$prep of 50 ms, on (ii) TI with fixed FA of $110^{\circ }$ and $T_{2}$prep of 50 ms, and on (iii) $T_{2}$prep pulse duration with fixed FA of $110^{\circ }$ and TI of 90 ms.

A high imaging FA of $110^{\circ }$ was chosen to achieve high SNR and good anatomical image quality at 0.55 T. For the bright-blood image, obtained directly from the 1st volume, the selected FA is a compromise between the near maximum value for arterial blood and the relatively low signals from the myocardium [Figure 2A(i)]. The FA beyond this value results in limited contrast gain compared to the quadratic increase in specific absorption rate (SAR) (12). Moreover, a slightly lower value of imaging FA is preferable for the black-blood contrast [Figure 2B(i)], which is generated from the direct magnitude subtraction of the 1st from the 2nd volume. A TI of 90 ms was employed to reduce both myocardial and epicardial fat signals while maintaining good signal from arterial blood in the 1st volume [Figure 2A(ii)]. A low dependence of TI on bight- and black-blood contrast values was observed [Figure 2A–B(ii)]. A pulse duration of 50 ms was used in the $T_{2}$prep-IR module to suppress the myocardial signal in the 1st volume [Figure 2A(iii)] and simultaneously to obtain a good signal on the subtracted image [Figure 2B(iii)]. Longer $T_{2}$prep generally yields lower SNR and is not beneficial for dictionary matching, which requires $T_{2}$prep durations below or close to the parametric values of the mapped myocardium tissue. Sufficient $T_{2}$ encoding is then provided by two $T_{2}$prep modules with different durations applied prior to the bSSFP acquisition, which also has intrinsic $T_{2}$-weighting. Although changing the order of $T_{2}$prep modules has almost no influence on the mapping sensitivity, it affects the contrast values of the anatomical imaging based on EPG simulations. For this reason, a shorter $T_{2}$prep of 30 ms was placed in the 3rd volume.

### 3.2 Phantom study

**Figure 3:**
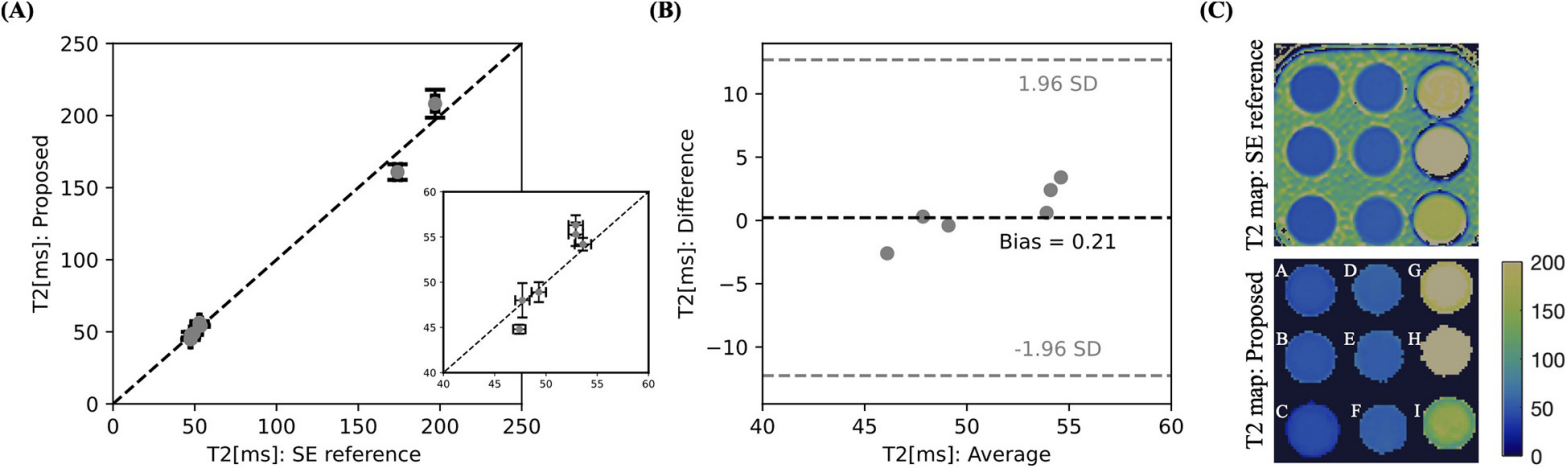
Validation of the proposed 3D $T_{2}$ multi-contrast mapping sequence in the standardized T1MES phantom with simulated HR of 60 bpm at 0.55 T against a 2D SE reference scan with correlation plot (**A**) and Bland-Altman analysis (**B**). The relevant $T_{2}$ myocardial region is zoomed in. (**C**) Phantom image of the proposed and reference sequences with the parametric values given in Table 1. The $T_{2}$ value for H vial was out of range and is therefore not shown.

Figure 3A shows good agreement in $T_{2}$ mapping with high linear correlation of $y=0.99x+0.28(R^{2}>0.99)$ between the proposed 3D approach and 2D SE references for a simulated HR of 60 bpm, whereby the H vial was excluded from the analysis due to unrealistically high $T_{1}$ at 0.55 T (see Table 1). From the Bland-Altman analysis (see Figure 3B), a minimal bias of 0.2 ms was obtained by averaging the signal from the first 8 imaging segments (≈25% of the central part of k-space).

### 3.3 In-vivo

**Figure 4:**
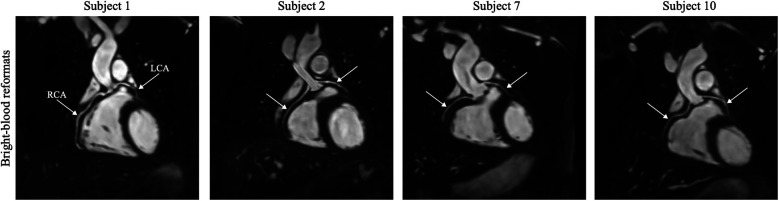
High-resolution bright-blood images are illustrated for four subjects in multiplanar reformation. The course of the left (LCA) and right (RCA) coronary arteries is indicated by white arrows.

**Figure 5:**
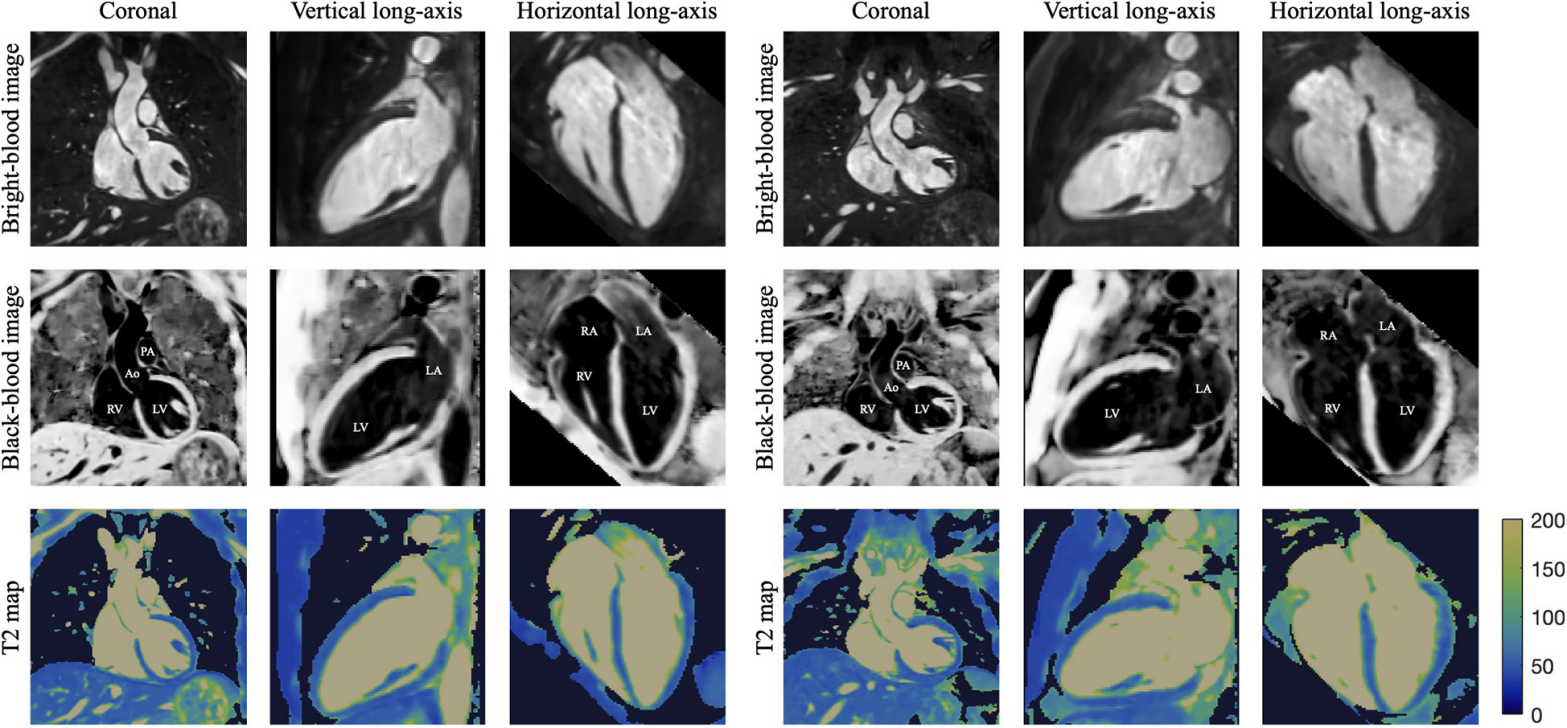
3D reconstructed data for two healthy subjects, showing bright- and black-blood images with co-registered $T_{2}$ maps in coronal, vertical long-axis (for evaluating the anterior/inferior walls and apex of the LV) and horizontal long-axis (for assessment of LV septal/lateral walls and apex, as well as RV free wall and chamber sizes) orientations. On the black-blood images different intrapericardial structures are indicated as: LV/RV—left/right ventricle, LA/RA—left/right atrium, Ao—ascending aorta, PA—pulmonary artery. To highlight relevant cardiovascular structures, a binary contrast-generated mask was applied to the $T_{2}$ map dataset.

**Figure 6:**
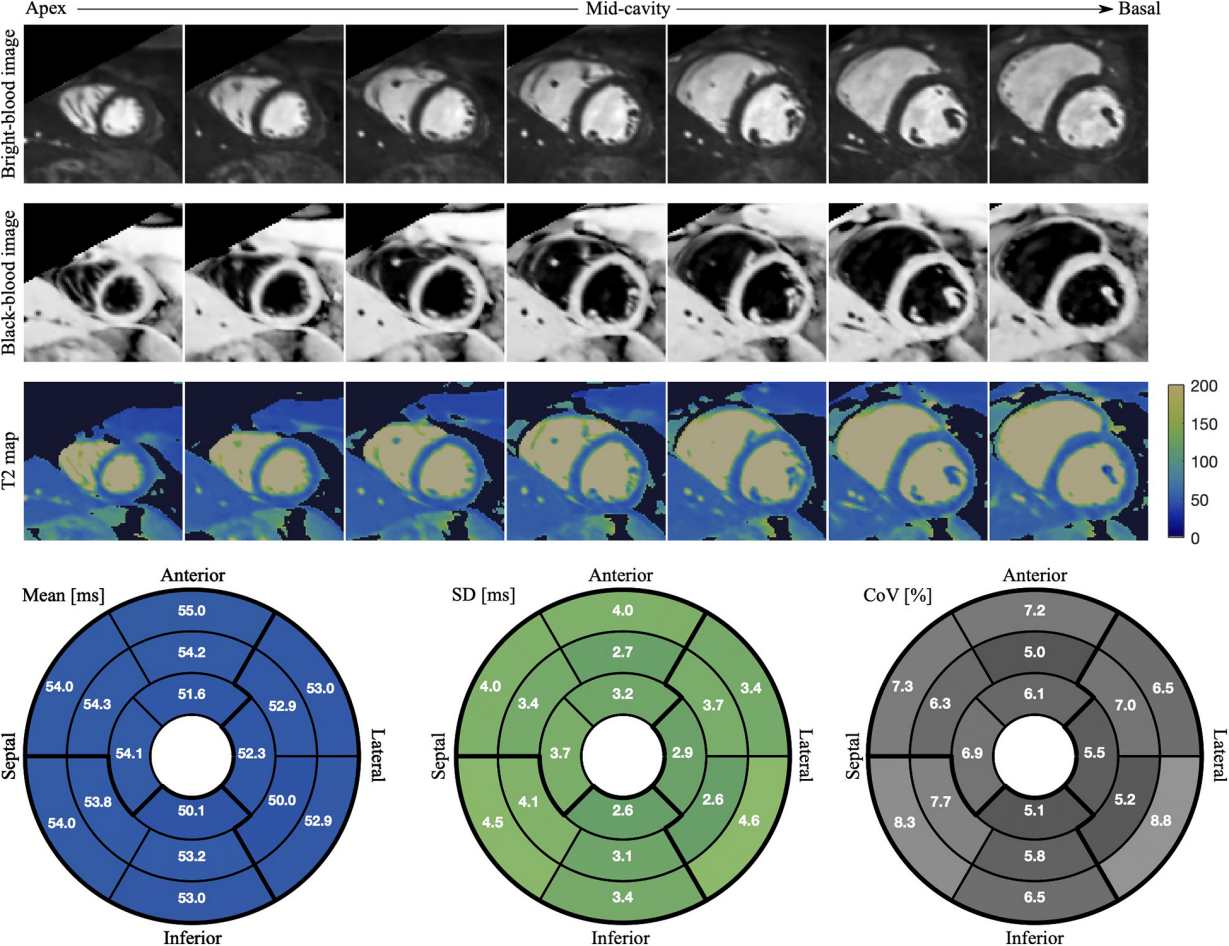
Bright- and black-blood images with co-registered $T_{2}$ maps of SAx cross-section slices from apex to base are displayed for a healthy subject with HR of 67 bpm. The mean $T_{2}$ values, SD and CoV in each of 16 segments are shown in the bull’s-eye-plot generated by projecting LV myocardial tissue onto a plane.

**Figure 7:**
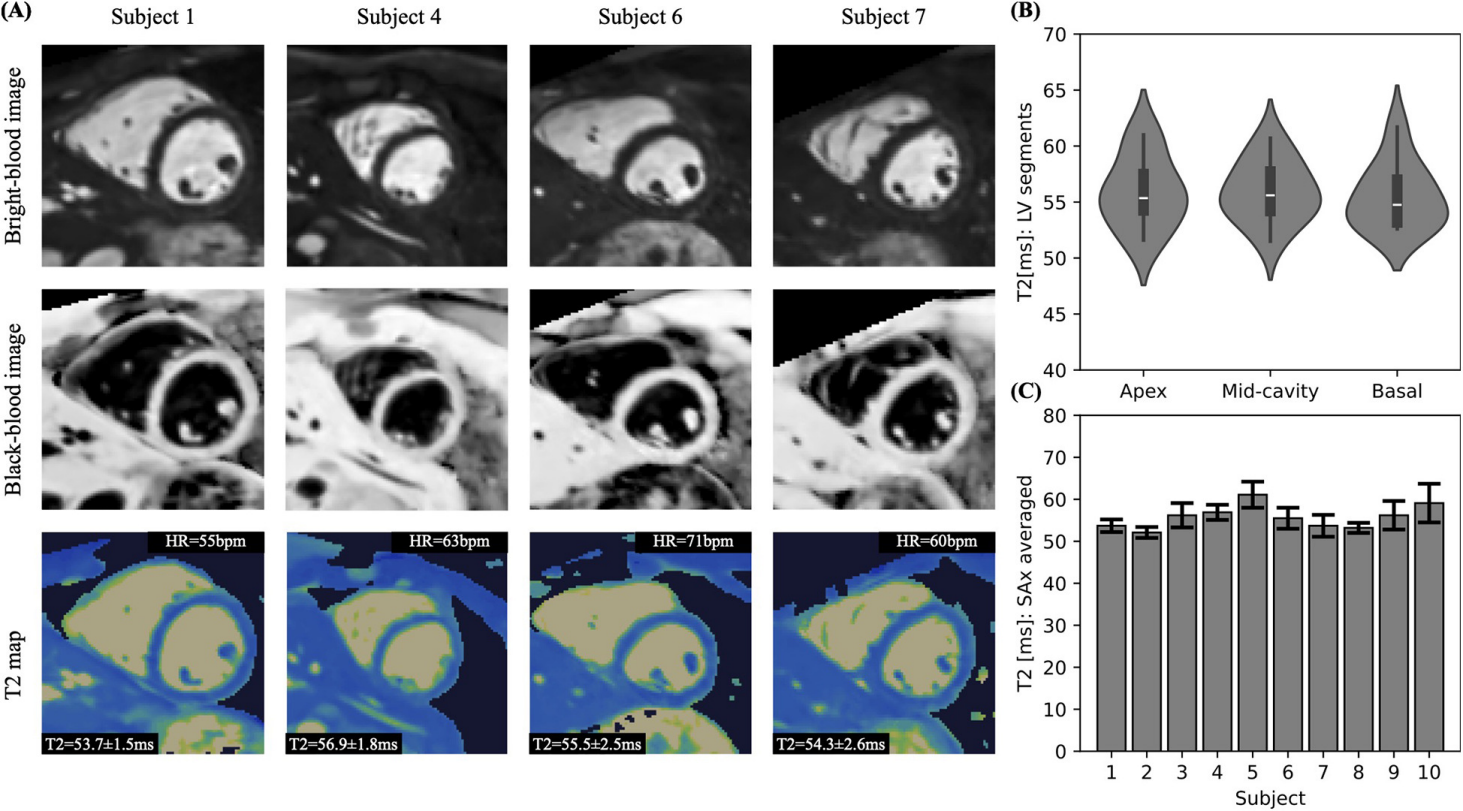
(**A**) Anatomical images and mapping shown for four representative subjects in mid-cavity SAx view. (**B**) Violin plot comparing the mean $T_{2}$ values from apical, mid-cavity and basal regions. (**C**) Comparison of $T_{2}$ mean and SD values across all subjects.

All experiments were successfully completed in healthy subjects with an average acquisition time of 8.5±0.9 min. One participant was excluded from statistical analysis due to highly variable HR and significant movement during scanning. Figure 4 illustrates reformatted bright-blood datasets to demonstrate the visibility of the left (LCA) and right (RCA) coronary arteries. Isotropic resolution of 1.5 mm allowed good depiction of the coronary arteries, including distal segments and major branches. The mean vessel sharpness of 58.6%±8.8% was obtained and the median image quality score was 3 (good: coronary artery adequately visualized and image of diagnostic quality). In Figure 5, 3D cardiovascular anatomy is displayed via bright- and black-blood imaging for two volunteers in coronal, VAx and HAx orientations along with $T_{2}$ maps. Excellent delineation of cardiac and vascular structures was observed together with spatially uniform mapping in the myocardium. Figure 6 displays SAx views from apex to base for a representative subject alongside with corresponding bull’s-eye-plots (30 slices), showing mean and SD $T_{2}$ values as well as CoV of the LV myocardium. In Figure 7A, bright- and black-blood images and corresponding $T_{2}$ maps are shown for four representative subjects in mid-ventricular SAx slice. Comparable $T_{2}$ values were measured in the septal segments across all volunteers (55.5±2.6)ms. Averaged $T_{2}$ values from all 3D datasets are displayed with violin plots: (55.9±3.2)ms (apex), (55.9±2.7)ms (mid-cavity), and (55.5±2.9) ms (basal) regions (see Figure 7B). The averaged $T_{2}$ values from all segments were (55.8±2.9) ms, which is consistent with literature value (13) (see Figure 7C).

## 4 Discussion

This study introduced a novel 3D whole-heart CMR sequence for simultaneous high-resolution anatomical imaging and myocardial tissue mapping at 0.55 T in an efficient and easy to plan 9 min scan. A unique sequence design allows multiple techniques to be combined in a single scan. The bright-blood volume is directly obtained from the first HB, while the black-blood volume is created by magnitude subtraction of the first from the second volume. Compared to the T2prep-IR BOOST sequence at 1.5 T (20), the preparation pulses were reordered to improve blood nulling on the directly subtracted images. As the native myocardial $T_{1}$ relaxation time is shorter at 0.55 T, a shorter inversion time was used compared to 1.5 T. The number of iNAV readouts was set to 6 instead of 14 (typical value at 1.5 T) due to faster recovery of epicardial fat at low-field, as shown in (24). Further adjustments were made to determine the most appropriate combination of $T_{2}$prep durations based on the prolonged $T_{2}$ at low-field. By matching the signal evolution with a previously generated EPG dictionary across all three volumes, co-registered $T_{2}$ maps were generated voxel-wise. The feasibility of the proposed sequence was demonstrated in a phantom study and in a cohort of healthy subjects. Precise and accurate $T_{2}$ values were observed in good agreement with the SE reference scan in the phantom study. In-vivo anatomical imaging of various intrapericardial structures received high-quality scores and $T_{2}$ maps provided comparable values across all subjects.

The acquisition of 3D volumes with isotropic resolution facilitated scan planning as cardiac orientations such as 2, 3 and 4 chamber and short-axis views can be obtained in a postprocessing step thereby minimizing scan complexity. The proposed sequence was implemented with in-line reconstruction, allowing the images to be viewed within approximately 3 min after the examination. To enable free-breathing acquisition, a 2D low-resolution iNAV was employed prior to each spiral interleave acquired per HB and used for respiratory binning and intrabin translational motion correction. The total scanning time is linearly related to the length of the acquisition window, the pixel size and the subject-dependent HR. Due to the high acceleration of 4 with the VD-CASPR trajectory, the 3D scan can be completed within a clinically feasible scan time of approximately 8 to 9 min. The acquisition window of the proposed sequence was kept short enough for the mid-diastolic application, a quiescent period of the cardiac cycle, resulting in less movement during the readout. Currently, the trigger delay is adjusted manually based on the CINE acquisition in the axial plane prior to the acquisition, which requires an additional planning step. To further improve image quality, the data is transferred from the scanner and denoised in a post-processing step using HD-PROST (28) in MATLAB, which takes about 10 min with current parameters. Generating one dictionary took less than a minute per subject and 3D $T_{2}$ maps were available in about two minutes afterwards. Due to the 3D nature and isotropic spatial resolution, the acquired images can be reformatted into any desired view to visualize whole-heart morphology and to analyse different intrapericardial structures. Bright-blood is the reference standard for imaging cardiac structures, great vessels and coronary arteries, whereas black-blood excels in visualizing the myocardium, atrial and vessel walls. The combination of both techniques improves the assessment of cardiac morphological evaluation compared to either technique in isolation. Complex coronary artery geometry prevents visualisation of the entire anatomy in a single slice, therefore multiplanar reformation was performed in coronal and axial views for the RCA and LCA, respectively. The chosen spatial resolution was deemed sufficient to visualise small structures such as the course and anatomy of the coronary arteries. Further reduction in voxel size would reduce the SNR, which may affect dictionary matching and thus precision of the estimated $T_{2}$ values.

The proposed sequence framework has several limitations. In the anterior segments of LV, the CoVs values are slightly increased possibly due to susceptibility artifacts at the heart-liver-lung interface. Because of the reduced LV-RV wall thickness in the apical septal segments, partial volume artifacts between the myocardium and blood pool are more pronounced, potentially resulting in elevated $T_{2}$ values. In subjects with HR variations, mapping accuracy and precision are expected to decrease due to deviations from the steady-state signal evolution assumed in the dictionary. Future work will include validation in patients with suspected cardiovascular disease. Whilst the preliminary experiments have shown good agreement between the $T_{2}$ values of the proposed sequence and the literature, further in-vivo validation against conventional 2D sequences is required for comparison. At the time the sequence was developed and implemented, there were no reference sequences available on the scanner for this purpose. To compensate for the loss of SNR and to obtain good image quality, a high FA of $110^{\circ }$ was used for bSSFP acquisition at 0.55 T, which is particularly challenging in the scanner used. The duration of the imaging RF pulses had to be extended to achieve higher FAs, which requires lower RF voltages (24). For higher FA the expected signal behaves more robustly to off-resonance phase accumulation due to $B_{0}$-inhomogeneity as shown in (34). One of the limitations is also that the $T_{2}$ estimate depends on the chosen fixed $T_{1}$, which may affect accuracy and precision in patients with $T_{1}$s that differ from normal myocardium [e.g., $T_{1}=760\;\mathrm{ms}$ “diseased” tissue vs. 700 ms for “healthy” tissue (13)]. To investigate its impact, additional dictionaries were generated with a coarse $T_{1}$ range of [600:50:800] ms. No significant statistical difference in $T_{2}$ was observed when mapping within the $T_{1}$ range of the additional dictionaries (see Supplementary Material). The high FA resulted in a reduced $T_{1}$ dependence of the sequence during bSSFP readout, as shown in (34), which is beneficial for $T_{2}$ mapping. Since $T_{2}$ elevation in the diseased myocardium is expected to increased by 10 ms to 20 ms, this effect is considered acceptable. Although the proposed sequence was designed to be less sensitive to longitudinal magnetization recovery, simultaneously acquired $T_{1}$ maps could not only improve the accuracy and precision of $T_{2}$ mapping but also provide complementary tissue characterization. In future studies, the sequence design will be expanded to include additional interleaves to enable simultaneous $T_{1}/T_{2}$ mapping.

## 5 Conclusions

In this work, we successfully demonstrated the feasibility of simultaneous whole-heart bright and black-blood anatomical imaging and $T_{2}$ mapping in phantoms and healthy subjects at 0.55 T in a single 9 min scan.

## Data Availability

The raw data supporting the conclusions of this article will be made available by the authors, without undue reservation.
