# Supplementary material for: High-resolution 3D whole-heart bright- and black-blood imaging with co-registered T2 mapping at 0.55 T
Source: Front Cardiovasc Med. 2025 Jul 3;12:1572318. doi: 10.3389/fcvm.2025.1572318 (PMC12267252; doi:10.3389/fcvm.2025.1572318)
Supplement: Supplementary file 4 [file Datasheet1.pdf]

Supplementary Material

1 SUPPLEMENTARY DATA

2 SUPPLEMENTARY TABLES AND FIGURES

2.1 Figures

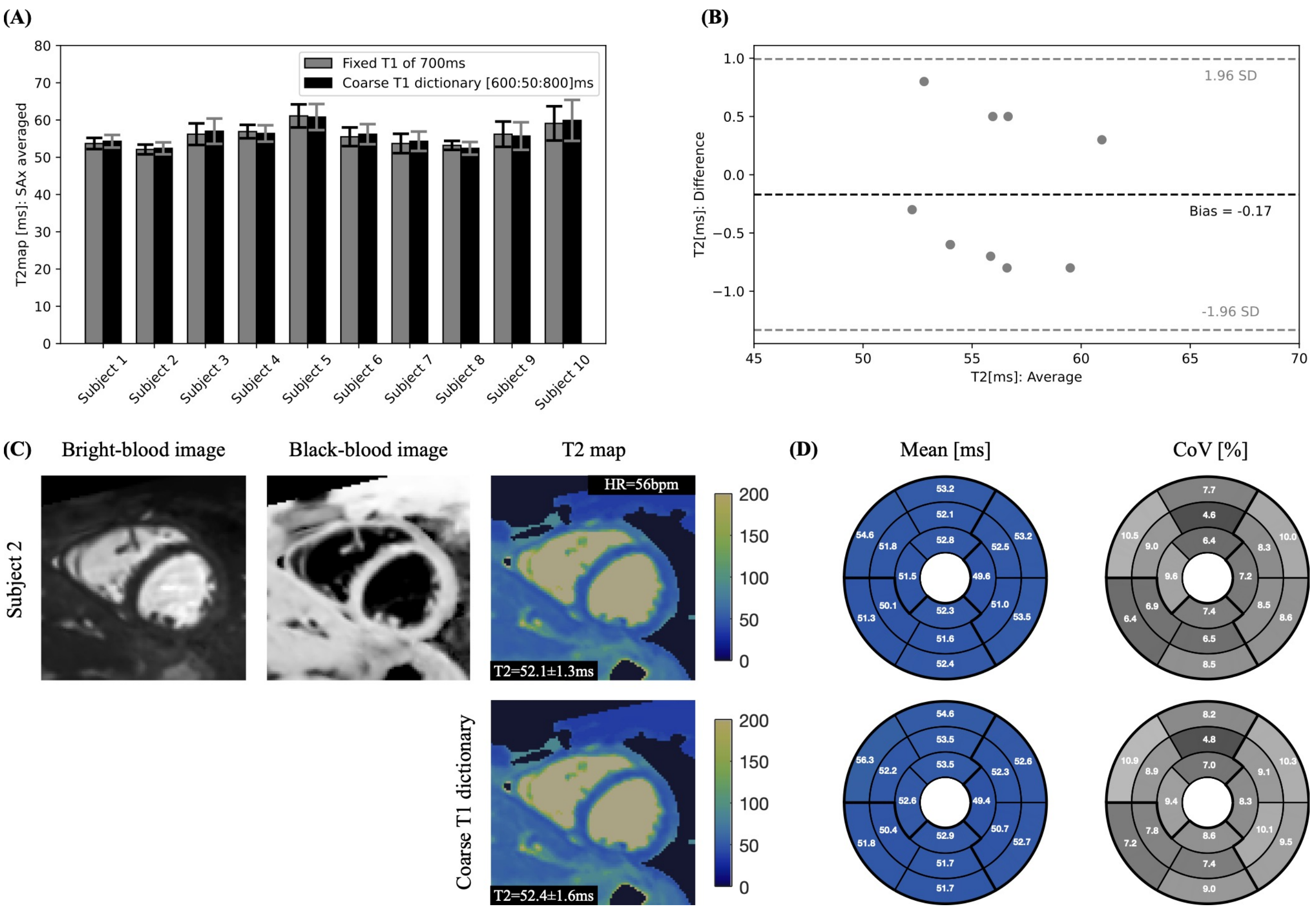

Figure S1. Comparison of averaged SAx T_2 values generated with fixed and coarse dictionary T_1 using bar chart (**A**) and Bland-Altman plot (**B**) across all subjects. **C**: Bright- and black -blood images with co-registered T_2 maps shown for a healthy subject using both methods without any visual difference. D: Bull's-eye plots for mean T_2 values and CoVs.
